# Retrospective Evaluation of Marker-Assisted Selection for Resistance to Bacterial Cold Water Disease in Three Generations of a Commercial Rainbow Trout Breeding Population

**DOI:** 10.3389/fgene.2018.00286

**Published:** 2018-08-03

**Authors:** Sixin Liu, Roger L. Vallejo, Jason P. Evenhuis, Kyle E. Martin, Alastair Hamilton, Guangtu Gao, Timothy D. Leeds, Gregory D. Wiens, Yniv Palti

**Affiliations:** ^1^National Center for Cool and Cold Water Aquaculture, Agricultural Research Service, United States Department of Agriculture, Kearneysville, WV, United States; ^2^Troutlodge, Inc., Sumner, WA, United States; ^3^Hendrix Genetics Aquaculture BV/Netherlands, Boxmeer, Netherlands

**Keywords:** rainbow trout, bacterial cold water disease, haplotype, SNP, MAS, QTL, Flavobacterium psychrophilum

## Abstract

Bacterial cold water disease (BCWD), caused by *Flavobacterium psychrophilum*, is an endemic and problematic disease in rainbow trout (*Oncorhynchus mykiss*) aquaculture. Previously, we have identified SNPs (single nucleotide polymorphisms) associated with BCWD resistance in rainbow trout. The objectives of this study were (1) to validate the SNPs associated with BCWD resistance in a commercial breeding population; and (2) to evaluate retrospectively the accuracy of MAS (marker-assisted selection) for BCWD resistance in this commercial breeding program. Three consecutive generations of the Troutlodge May breeding population were evaluated for BCWD resistance. Based on our previous studies, a panel of 96 SNPs was selected and used to genotype the parents and ten offspring from each of the 138 full-sib families of the 2015 generation, and 37 SNPs associated with BCWD resistance were validated. Thirty-six of the validated SNPs were clustered on chromosomes Omy3, Omy8 and Omy25. Thus, at least three QTL (quantitative trait loci) for BCWD resistance were validated in the 2015 generation. Three SNPs from each QTL region were used for haplotype association analysis. Three haplotypes, Omy3TGG, Omy8GCG and Omy25CGG, were found to be associated with BCWD resistance in the 2015 generation. Retrospective analyses were then performed to evaluate the accuracy of MAS for BCWD resistance using these three favorable haplotypes. The accuracy of MAS was estimated with the Pearson correlation coefficient between the total number of favorable haplotypes in the two parents and the family BCWD survival rates. The Omy8 and Omy25 haplotypes were positively correlated with the family BCWD survival rates across all three generations. The accuracies of MAS using these two haplotypes together were consistently around 0.5, which was equal or greater than the accuracy of the conventional family-based selection in the same generation. In conclusion, we have demonstrated that MAS for BCWD resistance is feasible in this commercial rainbow trout breeding population.

## Introduction

Bacterial cold water disease (BCWD), caused by *Flavobacterium psychrophilum*, is one of the most devastating diseases in rainbow trout (*Oncorhynchus mykiss*) aquaculture (Nematollahi et al., 2003; Starliper, 2011; Loch and Faisal, 2015). Licensed vaccines for BCWD are not available at present. Use of licensed antibiotics for BCWD treatment increases production costs, and antibiotic-resistant pathogens may emerge. Fortunately, genetic variation for BCWD resistance (Hadidi et al., 2008; Silverstein et al., 2009) has been documented in rainbow trout. Using family-based selection, a rainbow trout line with improved BCWD resistance has been developed (Leeds et al., 2010; Wiens et al., 2013a). However, family-based selection is based on the family breeding values estimated from pedigree relationships and BCWD phenotypes. Evaluation for BCWD resistance in rainbow trout is labor intensive and time consuming. Also, family-based selection cannot exploit within-family genetic variation because disease challenged individuals are excluded as selection candidates, and selection is based on family means obtained from the BCWD performance of siblings of the selection candidates. Thus, selection methods that can utilize within-family variation at an affordable cost are desirable.

Genomic selection (GS), which uses genetic markers covering the whole genome to calculate the genomic estimated breeding values of selection candidates, can improve the accuracy of selection in animal breeding (Meuwissen et al., 2013). Recently, we have demonstrated that GS can substantially increase the accuracy of selection for BCWD resistance in the Troutlodge May spawning population (Vallejo et al., 2017a). While the accuracy of family-based selection for BCWD resistance was 0.36, the accuracy of GS was as high as 0.71 in the same generation. To reduce the cost of genotyping, low-density marker panels were tested for GS. Surprisingly, we have recently found that the accuracy of GS using 70 SNPs (single nucleotide polymorphisms) associated with BCWD resistance is still better than the accuracy of conventional family-based selection in that commercial breeding population (Vallejo et al., 2018). This result indicates that marker-assisted selection (MAS) using only a few SNPs associated with the major QTL (quantitative trait loci) for BCWD resistance might further reduce the cost of genotyping.

It has been documented that MAS can improve the efficiency of selection of quantitative traits (Lande and Thompson, 1990). However, there are only few well-documented examples of MAS in aquaculture breeding programs, although many QTL for diverse traits in aquaculture species were reported in over a decade (Ashton et al., 2017). One of the best known examples is MAS for resistance to infectious pancreatic necrosis virus (IPNV) in Atlantic salmon (*Salmo salar*) (Houston et al., 2008; Moen et al., 2009, 2015; Gonen et al., 2015). QTL mapping revealed microsatellites associated with a major QTL for IPNV resistance in Atlantic salmon, and the microsatellites were used initially for MAS. Recently, the gene controlling for IPNV resistance has been identified (Moen et al., 2015), and two SNPs closer to the causative gene are used for commercial implementation of MAS for IPNV resistance in Atlantic salmon. Another successful example of MAS in aquaculture species is the development of Japanese flounder (*Paralichthys olivaceus*) resistant to lymphocystis disease (LD) (Fuji et al., 2007), an iridoviral disease found in many fish species. A major QTL for LD resistance in Japanese flounder was identified by microsatellite QTL mapping (Fuji et al., 2006). The microsatellite linked to the major QTL was used to develop a commercial population of Japanese flounder with no incidence of LD on farm trials while LD incidences were recorded in the control population. Recently, commercial rainbow trout eggs marketed as QTL-innOva® FLAVO (https://aquagen.no/en/), developed by MAS of two QTL for BCWD resistance, have been advertised. However, detailed information about these two QTL has not been published in the scientific literature.

In the past, we have reported several studies of identification of molecular markers associated with BCWD resistance in rainbow trout. Initially, microsatellites were used for QTL mapping, and nine QTL (Vallejo et al., 2013) were identified in the odd-year mapping families of the National Center for Cool and Cold Water Aquaculture (NCCCWA). Similarly, two major QTL (Wiens et al., 2013b) were identified in an even-year NCCCWA mapping family, and the QTL on chromosome Omy19 was validated in a later generation (Vallejo et al., 2014; Liu et al., 2015). Due to the limited number of microsatellites available for genetic mapping, restriction site associated DNA sequencing was later used to identify SNPs associated with the QTL for BCWD resistance (Liu et al., 2015; Palti et al., 2015b). Recently, a 57K SNP genotyping array has been developed in rainbow trout (Palti et al., 2015a), and a reference rainbow trout genome became available (GenBank Assembly Accession GCA_002163495). The development of those resources enabled a genome-wide association study to identify SNPs associated with BCWD resistance (Vallejo et al., 2017b) in two rainbow trout populations, the NCCCWA odd-year population and the 2013 Troutlodge May spawning population. Since the SNPs associated with BCWD resistance were discovered in different mapping populations, and several methods were used for QTL mapping, it is critical to validate the SNPs associated with BCWD resistance before implementation of MAS in a breeding program. The objectives of this study were (1) to validate the SNPs associated with BCWD resistance in the 2015 Troutlodge May spawning population; and (2) to evaluate retrospectively the accuracy of MAS for BCWD resistance in three consecutive generations of the Troutlodge May spawning population.

## Materials and methods

### Ethic statement

The experiments were conducted with the protocols approved by the Institutional Animal Care and Use Committee, National Center for Cool and Cold Water Aquaculture, Agriculture Research Service, United States Department of Agriculture. All efforts were made to minimize suffering and to ensure fish welfare.

### Three consecutive generations of the troutlodge may spawning strain

Troutlodge, Inc., has four rainbow trout strains (Liu et al., 2017) named by their peak spawning months. All four Troutlodge strains have been under selection for growth but not for BCWD resistance. Three consecutive generations of the odd-year Troutlodge May spawning strain were used in this study (Table 1). In a previous study (Vallejo et al., 2017b) we identified SNPs associated with BCWD resistance in the 2013 generation. In this study, 138 full-sib families in the 2015 generation were used to validate the SNPs associated with BCWD resistance. All three generations were used for retrospective evaluation of MAS for BCWD resistance. Fin clips were collected from all parents and offspring, and were stored in 95% ethanol. DNA was extracted from fin clips using an AutoGenprep 965 automated DNA extractor (Autogen, Holliston, MA, USA) following the manufacturer's instructions.

**Table 1:** Three consecutive generations of the odd-year Troutlodge May spawning rainbow trout strain used in this study.

**Generation**	**Type of study**	**No. of families**	**No. of dams**	**No. of sires**
2013	Discovery and MAS	101	101	62
2015	Validation and MAS	138	138	55
2017	MAS	120	120	65

### BCWD challenge experiments

In addition to the two BCWD challenges of the 2013 and 2015 generations described in our previous study (Vallejo et al., 2017a), 120 full-sib families of the 2017 generation were evaluated for BCWD resistance in this study. Eyed eggs of each family were provided by Troutlodge, Inc., and were hatched at the NCCCWA. At 80 days post-hatch, each family was challenged with BCWD following the established protocol described previously in detail (Hadidi et al., 2008). Briefly, 80 fish per family were stocked in two tanks with 40 fish per tank. The fish were challenged by intraperitoneal injection of *Flavobacterium psychrophilum* strain CSF259-93. Mortalities were collected daily for 21 days after intraperitoneal injection. Survival days (DAYS), the number of days to death post-challenge, were recorded. Survivors at the end of the experiment were assigned a value of 21 days. Each individual fish also had a record of survival status (STATUS). The binary trait STATUS had two classes: fish died during the 21 days period were assigned a value of 2, and fish alive on day 21 post-challenge were assigned a value of 1. The family BCWD survival rate was calculated as the percentage of survivors on day 21 post-challenge.

### Genotyping of the 2015 year-class population

SNP Type™ assays (Table S1) were developed for 96 SNPs associated with BCWD resistance identified in our previous studies (Liu et al., 2015; Palti et al., 2015b; Vallejo et al., 2017b). These 96 SNPs were used to genotype the 2015 population consisting of 138 full-sib families. Two parents and ten offspring of each 2015 family including the first five to die fish and five random survivors were genotyped following the protocol described in our previous study (Liu et al., 2016). Briefly, DNA samples were pre-amplified, and the pre-amplified products were diluted and used for genotyping with 96.96 Dynamic Array IFCs (Integrated Fluidic Circuits). The arrays were read using EP1 system, and genotypes were called automatically using Fluidigm SNP genotyping analysis software 4.1 with a confidence threshold of 85. The genotype clusters were examined by eye for each assay, and any wrong calls or no calls were corrected manually. The computer program PedCheck (O'connell and Weeks, 1998) was used to identify genotypes with Mendelian inheritance errors between parents and their offspring.

### Genotyping of parents of 2013 and 2017 populations

Parents of the 2013 generation were genotyped with the rainbow trout 57K SNP array as described in our previous publications (Palti et al., 2015a; Vallejo et al., 2017a). Briefly, genotyping was performed by a commercial genotyping service provider (GeneSeek/Neogen, Inc., Lincoln, NE), and the genotype data were filtered with our in-house bioinformatics pipeline to remove low quality genotypes. A customized Illumina SNP bead-array including 10K rainbow trout SNPs was used to genotype parents of the 2017 generation. The genotyping service for the Illumina array was also provided by GeneSeek/Neogen, Inc., Lincoln, NE.

### Family-based association analysis for BCWD resistance

The program PLINK 1.9 (Chang et al., 2015) was used for family-based association analysis to identify SNPs associated with BCWD resistance (*P* < 0.01) in the 2015 generation. The procedure QFAM was used to analyze the phenotypic data DAYS, and the PERM option was used to account for the dependence between related individuals. The procedure TDT (transmission disequilibrium test) was used to analyze the binary phenotype STATUS. SNPs with MAF (minor allele frequency) smaller than 0.05 were excluded from the association analyses.

Haplotype association analysis was performed using the program PLINK 1.07 (Purcell et al., 2007). Three SNPs (Table 2) from each QTL region were selected for haplotype analysis following these criteria: (1) they were associated with the BCWD resistance (see results); (2) their MAFs are larger than 0.2, which ensures that these SNPs are informative; and (3) they span a genomic region smaller than 2 Mb. The option—hap-impute was used to impute the haplotypes of parents and offspring of the 2015 generation, and the option—mhf 0.1 was used to filter out haplotypes with a frequency less than 0.1. The output files were then used to identify haplotypes associated with DAYS or STATUS (*P* < 0.01) using the same procedures described above to identify SNPs associated with BCWD resistance. The haplotypes associated with BCWD resistance are referred as favorable haplotypes hereafter.

**Table 2:** SNPs used for haplotype association analyses in the 2015 generation.

**Chromosome**	**Assay**	**SNP**	**Genomic position (bp)**	**Favorable allele^a^**	**Unfavorable allele**
3	P176	Affx-88951474	55254152	T	G
3	P161	Affx-88905010	55611964	G	T
3	P178	Affx-88960392	57059195	G	A
8	P194	Affx-88952288	76747151	G	A
8	P193	Affx-88952255	77402517	C	T
8	P191	Affx-88927298	78064599	G	A
25	P214	Affx-88927676	19553268	C	A
25	P212	Affx-88924886	20751780	G	T
25	P228	Affx-88947667	21146360	G	T

a *Allele associated with BCWD resistance (see Table 3)*.

### Evaluation of MAS for BCWD resistance

Haplotypes of the three QTL regions were reconstructed for all parents of the three generations using the default setting of program Beagle version 4.1 (Browning and Browning, 2007). The number of favorable haplotypes in each parent was counted for each QTL region. All the statistical analyses described below were performed using R version 3.4.3 (R Core Team, 2017). The accuracy of MAS was defined as the Pearson correlation coefficient between the total number of favorable haplotypes in the two parents and the family BCWD survival rates, similar to the approach we previously used for estimating accuracies of GS and traditional family-based selection (Vallejo et al., 2017a). The R function cor.test was used to test if the correlation coefficient is significantly different from 0 (*P* < 0.05). The families in each generation were grouped by the total number of favorable haplotypes in the two parents. After removing the haplotype groups with less than 10 families, analysis of variance was performed to test if the number of favorable haplotypes was associated with family BCWD survival rates. Then, R function TukeyHSD was used to compare the mean family BCWD survival rates between the haplotype groups.

### Accuracy of family-based selection for BCWD resistance

The estimated breeding values (EBV) of parents of the 2017 generation were estimated using BCWD survival phenotypes from the 2013 and 2015 disease challenges and pedigree records spanning eight generations. The records of BCWD survival STATUS were fit into pedigree-based BLUP (best linear unbiased prediction) threshold model using the computer program BLUPF90 (Misztal et al., 2015) as we have previously described (Vallejo et al., 2017a). The accuracy of family-based selection was estimated with the Pearson correlation coefficient between mid-parent EBV and the family BCWD survival rates.

## Results

### Validation of SNPs associated with BCWD resistance

Among the 96 SNP assays used to genotype the 2015 parents and offspring, six assays (Table S1) were removed from further analyses due to difficulties to score the genotypes or more than five Mendelian inheritance errors were identified between the genotypes of parents and their offspring. Eight SNPs (Table S1) were also removed from the association analyses because their MAFs were smaller than 0.05. Of the remaining 82 SNPs, 37 SNPs were associated with BCWD resistance in the 2015 generation (Table 3). These 37 SNPs were located on chromosomes Omy3, Omy8, Omy11 and Omy25. Since only one of the 37 SNPs was located on chromosome Omy11, and this SNP was only significant for STATUS, we focused on the other three chromosomes in the remaining analyses. For simplicity, we will refer to each of the three chromosomal regions as a QTL region hereafter.

**Table 3:** SNPs associated with BCWD resistance in the 2015 generation.

**Chromosome**	**Assay^a^**	**SNP**	**Genomic position (bp)**	**Allele 1**	**Allele 2**	**Effect^b^**	***P* (DAYS)**	**Odds ratio^c^**	***P* (STATUS)**
3	P174	Affx-88937637	54450618	A	*G*	-2.429	1.29E-03	NS^d^	NS
3	**P176**	Affx-88951474	55254152	G	*T*	-1.583	6.27E-03	NS	NS
3	**P161**	Affx-88905010	55611964	G	*T*	NS	NS	0.8059	3.57E-03
3	**P178**	Affx-88960392	57059195	G	*A*	2.978	1.00E-06	0.6351	2.89E-09
3	P177	Affx-88953526	57507731	C	*T*	-2.312	4.30E-03	NS	NS
3	P164	Affx-88913258	61729864	C	*T*	-1.877	2.70E-04	NS	NS
8	P184	Affx-88905931	NA^e^	T	*G*	-3.637	3.00E-06	NS	NS
8	P190	Affx-88924265	73174972	G	*A*	1.932	4.80E-04	NS	NS
8	P188	Affx-88921363	75663555	C	*T*	-2.992	1.00E-06	NS	NS
8	P195	Affx-88959850	76653343	G	*A*	-3.863	1.00E-06	NS	NS
8	**P194**	Affx-88952288	76747151	A	*G*	-3.171	1.00E-06	1.265	1.83E-03
8	P185	Affx-88910955	76881484	C	*A*	-4.11	1.00E-06	NS	NS
8	P187	Affx-88920166	77396013	A	*G*	-2.862	2.00E-06	NS	NS
8	**P193**	Affx-88952255	77402517	T	*C*	-2.327	1.00E-06	NS	NS
8	**P191**	Affx-88927298	78064599	A	*G*	-3.022	1.00E-06	NS	NS
8	P192	Affx-88948349	80284702	C	*T*	1.625	3.40E-04	NS	NS
11	P100	Affx-88938649	12692677	G	*T*	NS	NS	1.227	3.27E-03
25	P225	Affx-88940314	NA	G	*A*	3.611	1.00E-06	0.788	9.58E-04
25	P204	Affx-88907684	16542257	G	*A*	-3.423	1.00E-06	1.271	2.43E-03
25	P208	Affx-88913006	16886009	T	*C*	-2.36	2.30E-05	NS	NS
25	P226	Affx-88940680	17324440	A	*G*	1.833	3.68E-04	0.8193	7.93E-03
25	P242	RTRAD89nt123664	17799767	T	*C*	-3.56	1.00E-06	1.342	6.18E-05
25	P230	Affx-88956678	18192885	G	*T*	4.177	1.00E-06	0.6857	5.94E-06
25	P205	Affx-88909084	18318292	G	*T*	2.724	3.00E-06	0.8137	4.39E-03
25	P229	Affx-88953068	18886831	G	*A*	-2.044	4.77E-03	NS	NS
25	**P214**	Affx-88927676	19553268	C	*A*	3.769	1.00E-06	0.7014	1.48E-06
25	P232	Affx-88958660	20731488	T	*G*	-2.18	5.36E-03	1.354	1.18E-03
25	**P212**	Affx-88924886	20751780	T	*G*	-4.598	1.00E-06	1.652	1.62E-09
25	P224	Affx-88939492	21019355	C	*T*	-2.542	5.85E-05	1.288	9.34E-04
25	**P228**	Affx-88947667	21146360	T	*G*	-4.567	1.00E-06	1.566	2.98E-06
25	P222	Affx-88934536	21530601	A	*C*	4.549	1.00E-06	0.5455	2.64E-06
25	P217	Affx-88929795	21673784	A	*C*	-4.743	1.00E-06	1.573	2.99E-08
25	P231	Affx-88958425	22977788	C	*A*	-4.778	1.00E-06	1.395	3.07E-04
25	P206	Affx-88911339	24743445	T	*C*	4.05	1.00E-06	0.6336	1.05E-09
25	P221	Affx-88934507	25083318	C	*T*	3.618	1.00E-06	0.6538	7.48E-07
25	P223	Affx-88939140	25597437	A	*G*	-2.801	1.62E-03	NS	NS
25	P219	Affx-88931566	30261444	C	*T*	-3.971	4.43E-04	1.494	5.46E-03

a *Assays used for haplotype association analysis (Table 2) are highlighted in bold*;

b *positive number indicates that allele 1 increases the number of survival days, and negative number indicates that allele 2 increases the number of survival days*;

c *greater than 1 indicates that allele 1 increases the chance to die from BCWD, and less than 1 indicates that allele 2 increases the chance to die from BCWD*;

d *not significant (P > 0.01)*;

e *SNP cannot be mapped onto reference genome*.

### Haplotypes associated with BCWD resistance

Three SNPs (Table 2) from each QTL region were used for haplotype association analysis in the 2015 generation, and the results are presented in Table 4. For each QTL region, we identified one haplotype associated with BCWD resistance (increases BCWD survival days or lowers the chance to die from BCWD) and one haplotype associated with BCWD susceptibility (decreases BCWD survival days or increases the chance to die from BCWD). Since one of the goals of rainbow trout breeding is to improve BCWD resistance, we focused on the haplotypes associated with BCWD resistance and not the haplotypes associated with BCWD susceptibility. Consistent with the single SNP association analysis, the BCWD resistant haplotypes had the combination of the favorable SNP alleles associated with BCWD resistance (Table 3). To explicitly reference each haplotype, we added its chromosomal name ahead of its allele combination. Thus, the three haplotypes associated with BCWD resistance were referred to as Omy3TGG, Omy8GCG and Omy25CGG, respectively.

**Table 4:** Haplotypes associated with BCWD phenotypes in the 2015 generation.

**Chromosome**	**Haplotype**	**Effect^a^**	***P* (DAYS)**	**Odds ratio^b^**	***P* (STATUS)**
3	TGG	2.037	1.80E-05	0.6199	1.37E-10
3	GTA	−2.348	2.25E-04	NS^c^	NS
8	ATA	−3.553	2.00E-06	NS	NS
8	GCG	3.032	1.00E-06	0.7482	1.33E-04
25	ATT	−4.369	1.00E-06	1.617	1.37E-06
25	CGG	3.863	1.00E-06	0.7016	1.86E-06

a *Positive number indicates favorable haplotype which increases the number of survival days*;

b *less than 1 indicates that the haplotype lowers the chance to die from BCWD*;

c *not significant (P > 0.01)*.

### MAS for BCWD resistance

The haplotypes of each parent were reconstructed for each QTL region, and then we evaluated retrospectively the accuracies of MAS for BCWD resistance using the favorable haplotype for each QTL region (Table 5, Figure S1). Both haplotypes Omy8GCG and Omy25CGG were significantly correlated with the family BCWD survival rates across the three generations. However, haplotype Omy3TGG was only significantly correlated with the family BCWD survival rates in the 2013 generation. Thus, we decided to use the favorable haplotypes Omy8GCG and Omy25CGG together for MAS for BCWD resistance. The accuracies of MAS were consistently around 0.5 across the three generations (Table 5), which was higher than the accuracy of family-based selection (0.36) in the 2015 generation we reported previously (Vallejo et al., 2017a), and was similar to the accuracy of family-based selection (0.48) in the 2017 generation (Table 5).

**Table 5:** Accuracies of MAS and family-based selection in three generations of the odd-year Troutlodge May spawning strain.

**Generation**	**Omy3**	**Omy8**	**Omy25**	**Omy8 and Omy25**	**Three QTL**	**Family-based**
2013	0.26	0.34	0.45	0.52	0.52	NA^b^
2015	0.12^a^	0.21	0.46	0.48	0.45	0.36^c^
2017	0^a^	0.26	0.34	0.48	0.41	0.48

a *Correlation coefficient was not significant (P > 0.05)*;

b *data not available*;

c *data from Vallejo et al. (2017a)*.

**Figure 1:**
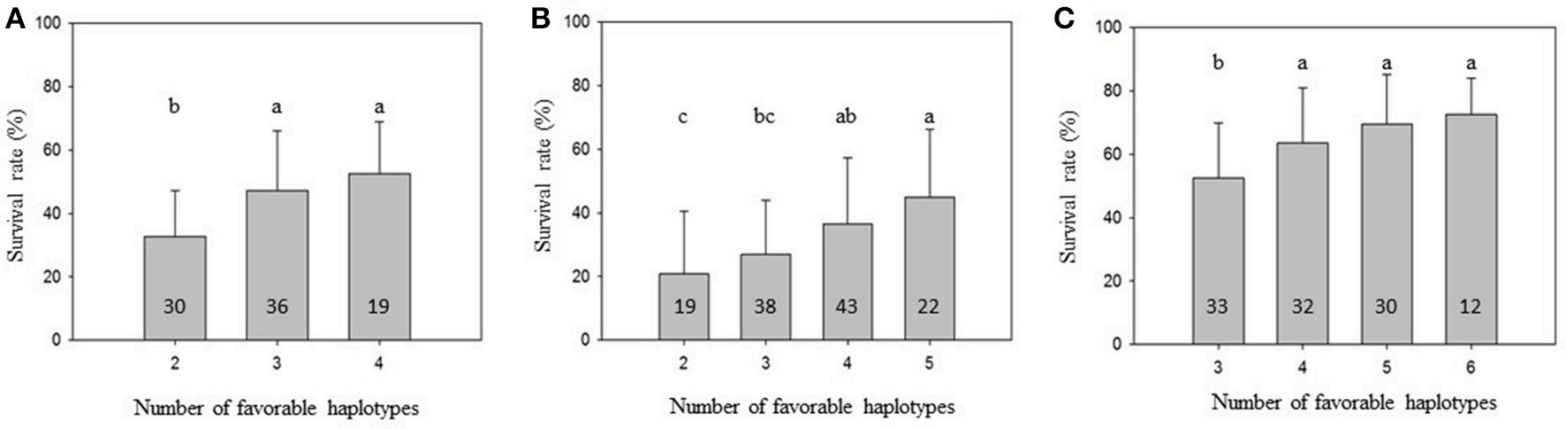
Mean family BCWD survival rate with an error bar. The families in each population were grouped by the total number of favorable haplotypes Omy8GCG and Omy25CGG in the two parents. Groups not sharing the same letter above the error bar have significantly different mean BCWD survival rates (Tukey's HSD, *P* < 0.05). The number in each column indicates the number of families in each haplotype group. **(A)** 2013 generation, **(B)** 2015 generation; **(C)** 2017 generation.

To further demonstrate that favorable haplotypes Omy8GCG and Omy25CGG can be used for MAS for BCWD resistance, we grouped the families by the total number of favorable haplotypes in the two parents. As shown in Figure 1, the mean family BCWD survival rates increased in all three generations with an increase in total number of favorable haplotypes in the two parents. The families with the highest number of favorable parental haplotypes had a significantly higher mean family BCWD survival rate than the families with the least number of favorable parental haplotypes in each generation. The magnitude of difference in percentage points was 19.8, 24.2 and 20.1 for 2013, 2015 and 2017 generations, respectively.

## Discussion

BCWD is one of the most devastating diseases in rainbow trout aquaculture. Previously, we have identified SNPs associated with BCWD resistance in rainbow trout (Liu et al., 2015; Palti et al., 2015b; Vallejo et al., 2017b), although we have found that QTL and SNPs associated with BCWD resistance can vary between populations. In this study, 37 SNPs associated with BCWD resistance were validated in the 2015 year-class population, and we have demonstrated that MAS for BCWD resistance in rainbow trout is feasible in three consecutive generations of the odd-year Troutlodge May spawning strain.

### Validation of QTL for BCWD resistance in rainbow trout

It is essential to validate the genetic markers associated with the traits of interest before implementation of MAS in breeding programs. Among the 96 SNPs associated with BCWD resistance identified in our previous studies (Liu et al., 2015; Palti et al., 2015b; Vallejo et al., 2017b), 37 SNPs were validated in the 2015 generation in this study. All validated SNPs, except one, were clustered on chromosomes Omy3, Omy8 and Omy25. Thus, at least three QTL for BCWD resistance were validated in the 2015 year-class population. This result is not unexpected because these three QTL had the largest effects in the 2013 year-class population (Vallejo et al., 2017b). Although 10 SNPs on chromosome Omy19 were included in the SNP panel, none of them was associated with BCWD resistance in the 2015 year-class population. This result is not surprising either because the QTL on Omy19 was identified and validated in even-year NCCCWA families (Wiens et al., 2013b; Vallejo et al., 2014; Liu et al., 2015). This result is also consistent with previous reports of population specific QTL for BCWD resistance (Vallejo et al., 2013, 2017b; Campbell et al., 2014; Palti et al., 2015b). Certainly, some QTL identified in our previous studies could be false positive, which might be another reason that some SNPs could not be validated in this study.

### Molecular breeding for BCWD resistance in rainbow trout

In this report, we have demonstrated through retrospective analysis that MAS for BCWD resistance is feasible in three consecutive generations of the odd-year Troutlodge May spawning strain. Three SNPs from each of the two major QTL regions on chromosomes Omy8 and Omy25 were used to reconstruct the haplotypes of all parents for the three consecutive generations. The mean family BCWD survival rates increased in all three generations with the increase of the total number of favorable haplotypes in the two parents. The estimated accuracies of MAS using these two favorable haplotypes together were consistently around 0.5 across all three generations of the odd-year Troutlodge May spawning strain, which was equal or greater than the accuracy of traditional family-based selection in the same generation. We are currently evaluating whether these SNPs and haplotypes are also associated with BCWD resistance in other rainbow trout aquaculture strains. Campbell et al. (2014) identified 12 SNPs associated with BCWD resistance in another commercial rainbow trout population. But, none of the 12 SNPs was located on chromosome Omy25, and the only significant SNP on chromosome Omy8 was located in a region different from the Omy8 QTL targeted in this study. Thus, MAS with the favorable haplotypes reported in this study may not work in the population used by Campbell et al. (2014).

MAS for a minor QTL may not be productive although successful examples of MAS for major QTL in aquaculture species were reported (Houston et al., 2008, 2012; Moen et al., 2009). We previously reported that the QTL on chromosome Omy3 was not a major QTL explaining only up to 2% of the genetic variance for BCWD resistance in the 2013 year-class population (Vallejo et al., 2017b). In this study, six SNPs on chromosome Omy3 were associated with BCWD resistance in the 2015 year-class population, and a haplotype on Omy3, associated with BCWD resistance, was also identified in the 2015 year-class population. However, the effect of this QTL was not as large as the other two QTL on chromosomes Omy8 and Omy25. The total number of Omy3 favorable haplotypes in the two parents was not significantly correlated with the family BCWD survival rates in both the 2015 and 2017 generations. In the 2013 generation, the correlation coefficient for this minor QTL was significant, but the accuracy of MAS remained the same with or without this Omy3 QTL (Table 5).

The successful MAS reported in this study provides a less expensive alternative for molecular breeding for BCWD resistance in rainbow trout. Previously, we have demonstrated that GS can substantially improve the accuracy of selection for BCWD resistance in rainbow trout (Vallejo et al., 2017a). The accuracy of GS for BCWD resistance in rainbow trout can be as high as 0.71 in the 2015 generation (Vallejo et al., 2017a), which is higher than the accuracy of MAS reported in this study. However, those results were based on one generation of GS, and the accuracy of GS depends on many factors such as experimental design, size of reference population, number of SNPs, and statistical models used to estimate the SNP effects (Vallejo et al., 2017a). Comparing to GS, MAS for BCWD resistance has several advantages. (1) The genotyping cost for MAS is much lower. Only six SNPs were used for retrospective MAS in this study. Thus, the genotyping cost of MAS is considerably lower than that of GS, which typically requires tens of thousands SNPs covering the whole genome. Recently, we found that the accuracy of GS for BCWD resistance in the 2015 generation with only 70 of the 96 SNPs used in this study can remain as high as that of the 57K SNP array (Vallejo et al., 2018). Nonetheless, the genotyping cost with six SNPs is still cheaper than that with 70 SNPs. (2) MAS, once established, does not require a reference population, and is solely based on the genotypes of candidate breeders. GS requires both genotypes and phenotypes from a reference population (or training sample) in each generation of selection. In addition to the added cost of genotyping the reference population, collecting BCWD phenotypes is labor intensive and requires a special fish challenge facility due to biosecurity constraints. (3) MAS can be used to directly select egg production fish to produce customized eggs with improved BCWD resistance. Genotyping of a large number of egg production fish for genomic selection is cost prohibitive and very complicated for logistical reasons. Therefore, the choice to implement MAS or GS for BCWD resistance in breeding programs depends on many factors including the breeding program goals, the resources available, the anticipated economic gains from the genetic improvement and the options available for maximizing the economic gains.

### Comparison of MAS and family-based selection for BCWD resistance

The accuracy of MAS was equal or greater than the accuracy of family-based selection in the same generation. The accuracy of MAS in the 2015 generation was 0.48, which was higher than 0.36, the accuracy of family-based selection in the same generation reported in our previous study (Vallejo et al., 2017a). The accuracies of selection in the 2017 generation were 0.48 for both MAS and the conventional family-based selection. The improved accuracy of family-based selection in the 2017 generation was mainly because BCWD phenotypes in two generations, 2013 and 2015, were available and used to estimate EBV of parents for the 2017 generation. Although there was not a large difference between these two methods in terms of the estimated accuracy of selection, MAS might still be more attractive in practice because MAS does not require obtaining BCWD phenotypes in each generation. However, the extent of improvement on BCWD resistance with MAS alone could be limited because only the two major QTL for BCWD resistance were targeted for MAS in this study. We reported previously that both major and minor QTL control BCWD resistance in the odd-year Troutlodge May spawning strain (Vallejo et al., 2017b). To maximize and sustain the genetic improvement for BCWD resistance through selective breeding over multiple generations, it would be necessary to integrate MAS and family-based selection to select both major and minor QTL for BCWD resistance in this commercial population.

## Conclusion

The accuracies of MAS for BCWD resistance using SNP haplotypes from two major QTL regions in the odd-year Troutlodge May spawning strain were consistently around 0.5 across three generations, which was equal or greater than the accuracy of conventional family-based genetic merit predictions in the same generation. Therefore, we have demonstrated in this report that MAS for BCWD resistance in rainbow trout is feasible in this commercial breeding population.

## Author contributions

YP and SL conceived and planned the study; YP, JE, TL and GW coordinated, supervised, and performed the disease challenge experiments; RV calculated the estimated breeding values of parents of 2017 generation; GG involved in the selection of SNPs used in this study; KM provided eyed eggs for all three Troutlodge populations and fin clips of all parents; AH provided the genotypes of parents of 2017 generation; SL performed the data analysis and drafted the manuscript. All authors read and approved the final manuscript.

### Conflict of interest statement

KM was employed by Troutlodge, Inc., and author AH was employed by Hendrix Genetics Aquaculture BV/Netherlands. The remaining authors declare that the research was conducted in the absence of any commercial or financial relationships that could be construed as a potential conflict of interest.
